# Low-light image enhancement using generative adversarial networks

**DOI:** 10.1038/s41598-024-69505-1

**Published:** 2024-08-09

**Authors:** Litian Wang, Liquan Zhao, Tie Zhong, Chunming Wu

**Affiliations:** Key Laboratory of Modern Power System Simulation and Control and Renewable Energy Technology(Ministry of Education), Northeast Electric Power University, Jilin, 132012 China

**Keywords:** Generative adversarial networks, Nighttime road scene image enhancement, Illumination attention module, Multi-scale feature extraction module, Biogeochemistry, Environmental social sciences, Environmental impact, Information technology

## Abstract

In low-light environments, the amount of light captured by the camera sensor is reduced, resulting in lower image brightness. This makes it difficult to recognize or completely lose details in the image, which affects subsequent processing of low-light images. Low-light image enhancement methods can increase image brightness while better-restoring color and detail information. A generative adversarial network is proposed for low-quality image enhancement to improve the quality of low-light images. This network consists of a generative network and an adversarial network. In the generative network, a multi-scale feature extraction module, which consists of dilated convolutions, regular convolutions, max pooling, and average pooling, is designed. This module can extract low-light image features from multiple scales, thereby obtaining richer feature information. Secondly, an illumination attention module is designed to reduce the interference of redundant features. This module assigns greater weight to important illumination features, enabling the network to extract illumination features more effectively. Finally, an encoder-decoder generative network is designed. It uses the multi-scale feature extraction module, illumination attention module, and other conventional modules to enhance low-light images and improve quality. Regarding the adversarial network, a dual-discriminator structure is designed. This network has a global adversarial network and a local adversarial network. They determine if the input image is actual or generated from global and local features, enhancing the performance of the generator network. Additionally, an improved loss function is proposed by introducing color loss and perceptual loss into the conventional loss function. It can better measure the color loss between the generated image and a normally illuminated image, thus reducing color distortion during the enhancement process. The proposed method, along with other methods, is tested using both synthesized and real low-light images. Experimental results show that, compared to other methods, the images enhanced by the proposed method are closer to normally illuminated images for synthetic low-light images. For real low-light images, the images enhanced by the proposed method retain more details, are more apparent, and exhibit higher performance metrics. Overall, compared to other methods, the proposed method demonstrates better image enhancement capabilities for both synthetic and real low-light images.

## Introduction

Image acquisition devices struggle to capture enough light under low-light conditions, leading to poor image quality. This directly impacts the effectiveness of nighttime traffic monitoring, the reliability of automated driving systems, and the clarity of dashcam footage^1^. Therefore, improving the quality of nighttime images is of significant importance. Enhancing the quality of low-light images can be achieved through improving road illumination, enhancing sensor performance, and optimizing image processing^2^. However, improving road illumination and sensor performance is costly and may face practical limitations. In contrast, optimizing nighttime road images is relatively low-cost and more accessible to apply on a large scale. Existing nighttime image enhancement methods can be broadly categorized into traditional methods, such as histogram equalization^3^ and Retinex-based methods^4^, and deep learning-based enhancement methods. Although traditional methods have developed relatively maturely, the continuous advancement of GPU technology has led to the widespread application of various convolutional neural networks (CNNs) in the field of image enhancement^5^. Deep CNNs offer superior performance and stronger generalization capabilities compared to traditional methods, making them more suitable for practical scenarios. To further improve the effectiveness of low-light enhancement based on deep convolutional neural networks, a new generative adversarial network is proposed for enhancing low-light images.

The main contributions of this paper include the following:

- We design a new generator network to improve low-light image quality. First, an illumination attention module is designed to enable the network to focus on crucial lighting features for accurate information extraction. Second, a multi-scale feature extraction module is designed to capture richer information by extracting features from various scales. Finally, a generator network with an encoder-decoder structure is designed by integrating these modules to enhance low-light images.
- A discriminative network with a dual-discriminator structure is designed to enhance the discriminative ability towards input images. It can indirectly improve the image generation performance of the generative network. This discriminative network comprises a local discriminator and a global discriminator. The local discriminator is used to determine the local brightness parts, while the global discriminator is used to determine the overall background information of the images.
- An improved loss function is proposed by introducing color loss and perceptual loss into the conventional loss function. The improved loss function can more accurately evaluate the degree of color distortion in the images, thereby reducing color distortion in the enhanced images.

## Related work

The methods for enhancing low-light nighttime images are mainly divided into traditional and deep learning methods. Traditional methods include histogram-based methods^3^ and Retinex-based methods^6^. Histogram equalization improves image quality by redistributing pixel values but may lead to local contrast imbalance and excessive emphasis on image background^7^. Recent advancements include methods based on fuzzy logic^8^and incorporating multiscale Retinex^9^, but issues such as color deviation and insufficient preservation of texture details persist. The traditional Retinex algorithm considers the internal attributes of images, enhancing image quality by decomposing and recomposing light in input images. However, this method is computationally complex, sensitive to noise, and difficult to handle complex scenes. Recently, Wu et al.^10^ proposed a regularization model using network inference to replace the manual prior model of the Retinex method. Yi et al.^11^ introduced an interpretable generative diffusion model incorporating forward and backward processes. Pan et al.^12^ devised a novel retinal method to improve illumination maps. However, the results of these enhancement methods are not entirely satisfactory and may sometimes produce additional artifacts. Additionally, there are other enhancement methods. For example, methods based on fractional-order denoising and multi-scale decomposition wavelet transforms^13^, channel-aware methods^14^, and contrast adjustment and fusion methods^15^.

Deep learning-based methods have demonstrated efficacy in the domain of low-light image enhancement. The deep learning approach employs a variety of one-to-one mapping relationships to enhance image quality. One advantage of deep learning is that many datasets provide the model with greater generalization ability. Yang et al.^16^ recently developed an end-to-end Retinex mapping network. The network adjusts the output of the decomposition sub-network by applying a sparse gradient constraint method. The network is capable of retaining the essential edge information while eliminating the low-amplitude interference present in the image. It is important to note that the network is not capable of accurately recovering images with extremely low luminance. Chen et al.^17^ proposed a new Retinex network for estimating illumination. The method enhances the original decomposition network and incorporates an illumination enhancement module to facilitate light recovery. While the method demonstrates satisfactory performance in luminance recovery, it fails to address the issue of local overexposure, which may arise. Ahn et al.^18^ proposed a learning-based Retinex network. The method initially smooths the light by edge preservation and overcomes the limitation of low light through a designed gamma correction module. Nevertheless, this method is not effective in preserving image texture details. Rahman et al.^19^ proposed a combined model to address the problem of uneven exposure in low-light images. This method first designs a convolutional neural network to classify the images. Then, it obtains the initial transmission map using the prior bright channel, which is improved with the L1 norm. Finally, the enhancement results are achieved by applying an image degradation model. Hai et al.^20^ proposed a novel Retinex-based real-low to real-normal network (R2RNet). This network’s three sub-networks decompose low-light images, reduce interference, and use spatial information of low-light images to improve contrast. However, the method also neglects the preservation of detailed information. Li et al.^21^ proposed an attention enhancement network with modular stacking. Firstly, image features are extracted and fused using a modular stacking method, after which the feature map is enhanced using a designed convolutional neural network (CNN). However, this method may also incorporate other disturbances during the enhancement process. Guo et al.^22^ proposed a new reference-free curve estimation method called Zero-DCE. This method employs a trained deep network to dynamically adjust the image, obviating the need for paired data. This reduces the risk of overfitting. However, the method is susceptible to overexposure. Subsequently, Li et al.^23^ proposed Zero-DCE++ as a means of optimizing the results of the preceding generation model. The method enhances the quality of the enhancement results while the proposed model is more lightweight and faster than the previous version. Nevertheless, the issue of overexposure persists. Ma et al.^24^ proposed a self-calibrating illumination (SCI) learning framework. A cascaded illumination learning module is initially employed for enhancement purposes, after which a weight-sharing module and a designed self-calibration module are utilized to achieve different stages of convergence. Nevertheless, this self-calibration approach is susceptible to color distortion.

At present, Generative Adversarial Networks (GANs) are employed in a multitude of fields, including super-resolution reconstruction^25^, image fusion^26^, and image denoising^27^. Furthermore, they demonstrate satisfactory outcomes. GANs comprise generators and discriminators that are trained through adversarial learning to generate images that are more closely aligned with the actual results^28^. Liu et al.^29^ proposed a Generative Adversarial Network (GAN) aimed at enhancing image details. This method utilizes ZeroDCE for initial image processing, followed by a specially designed U-Net for detail enhancement and noise suppression. Although this approach effectively preserves detailed information, the use of ZeroDCE as a preprocessing step introduces a minor color bias. Jiang et al.^30^ presented an enhancement method based on GANs. Their approach introduces a self-attention map to facilitate the training of unpaired datasets in an unsupervised manner. However, the generated images often exhibit small artifacts. Jia et al.^31^ proposed a cycle-consistent generative adversarial network (GAN). The generative network adopts an encoder-decoder architecture, incorporating a contextual feature extraction module and a receptive field residual module. The designed multi-scale discriminator network helps to fuse low-frequency and high-frequency information. Additionally, a new loss function was introduced. Fu et al.^32^ proposed an unsupervised low-light image enhancement network. The network was designed with a perceptual attention module, which enhances the network’s feature extraction. A novel identity-invariant loss function was developed to address the issue of overexposure. Although this method can obtain enhanced results, it is less effective in enhancing low-quality input images. Xu et al.^33^ proposed a low-light enhancement method based on generative adversarial networks. The method devised a novel objective function to facilitate network training and a novel feature extraction module to assist the network in recovering high-frequency information. However, the image enhanced by this network exhibits partial blurring. Jin et al.^34^ proposed a progressive transmission network (ProGAN )based on generative adversarial networks. Firstly, the image is decomposed using the JieP method of the Retinex model, and the relationship between the reflected component of the low-light image and the normal-light image is understood by the network reflection decomposition. Subsequently, another illumination transmission network (IllumTransN) is employed to transmit the light component of the normal-light image to the reflection component for low-light enhancement. Nevertheless, this network is susceptible to color distortion. Fu et al.^35^ proposed a generative adversarial network enhancement method for luminance attention. The modified method predicts the light distribution of an image by luminance attention and constructs a new generator based on the U-Net architecture. It is important to note that the method only considers the effect of light intensity on the enhancement of low-light images. Zhang et al.^36^ proposed a hybrid network based on Retinex and Generative Adversarial Networks. Retinex is responsible for the separation of reflective and illuminated components, and the GAN is responsible for helping to pair-train the network and eliminate interference. It is important to note that this method is not optimal for enhancing the shadow portion of low-light images. Sun et al.^37^ proposed a CycleGAN-based method for paired nighttime image enhancement. The method employs a CycleGAN for paired dataset training and a single-scale luminance transform method to enhance local contrast. The method places greater emphasis on the accurate representation of the enhanced image, yet it fails to address the issue of color distortion.

Existing models for enhancing low-light images mainly suffer from issues of overexposure and color distortion. They also struggle to preserve the texture details of the original images. To address these problems, we propose a novel generative adversarial network (GAN). The illumination attention module focuses on lighting features to prevent overexposure in the enhanced images. The multi-scale feature extraction module aids the network in extracting richer information, preserving the texture details and color information in the enhanced images. Additionally, the color loss helps the network better restore the colors in the enhanced images, reducing color distortion.

## Proposed methods

We propose a Generative Adversarial Network (GAN) method to enhance the quality of low-light images. This network consists of two components: the generative network and the discriminative network. The generative network aims to enhance low-light images to improve image quality. The discriminative network primarily distinguishes between real normal illuminated images and enhanced images. It can improve the image enhancement capability of the generative network through competition and cooperation between the generative and discriminative networks. In the following sections, we will provide detailed introductions to the generative network, the discriminative network, and the loss function separately.

### Proposed generative network

**Figure 1:**
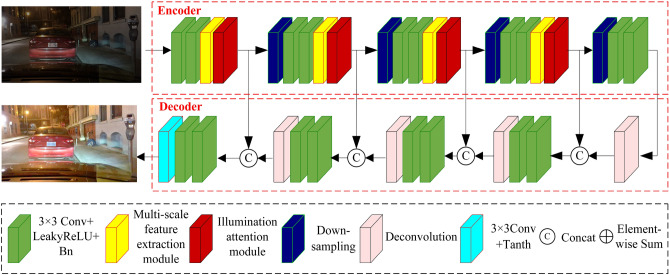
Proposed generative network.

As illustrated in Fig. 1, our designed generative network comprises an encoder and a decoder. In the encoder, we first design a basic feature extraction module, which consists of two consecutive 3$\times$3 convolutional layers (each followed by LeakyReLU activation function and BN layer), a multi-scale feature extraction module, and an illumination attention module. The encoder is divided into five parts: the first part includes a basic feature extraction module, and parts two to four consist of one downsampling module and one basic feature extraction module each. The fifth part comprises one downsampling operation and two consecutive 3$\times$3 convolutional layers (each followed by the LeakyReLU activation function and BN layer). The two consecutive 3$\times$3 convolutional layers in the basic feature extraction module are used for preliminary feature extraction of low-light images. The multi-scale feature extraction module can extract features from different scales, capturing detailed features at different scales. The illumination attention module dynamically adjusts the weights of different regions of the feature maps in both channel and spatial dimensions, enabling the network to focus more on important lighting features. The downsampling operation in the encoder facilitates further extraction of low-frequency information from images and increases the receptive field.

The decoder consists of one deconvolutional layer, three fusion modules with identical structure, two consecutive 3$\times$3 convolutional layers (each followed by LeakyReLU activation function and BN layer), and one 3$\times$3 convolutional layer with a tanh function. Each fusion module comprises two consecutive 3$\times$3 convolutional layers and one deconvolutional layer. The deconvolution operation increases the size of the feature maps, gradually restoring the feature map size through four deconvolutions. To preserve more spatial and contextual information, we adopt four skip connections, transmitting the feature maps from different depths of the encoder to the decoder and merging the corresponding feature maps through concatenation operations. Finally, we reconstruct high-quality low-light images from the fused features by using a 3$\times$3 convolutional layer with a tanh activation function.

**Figure 2:**
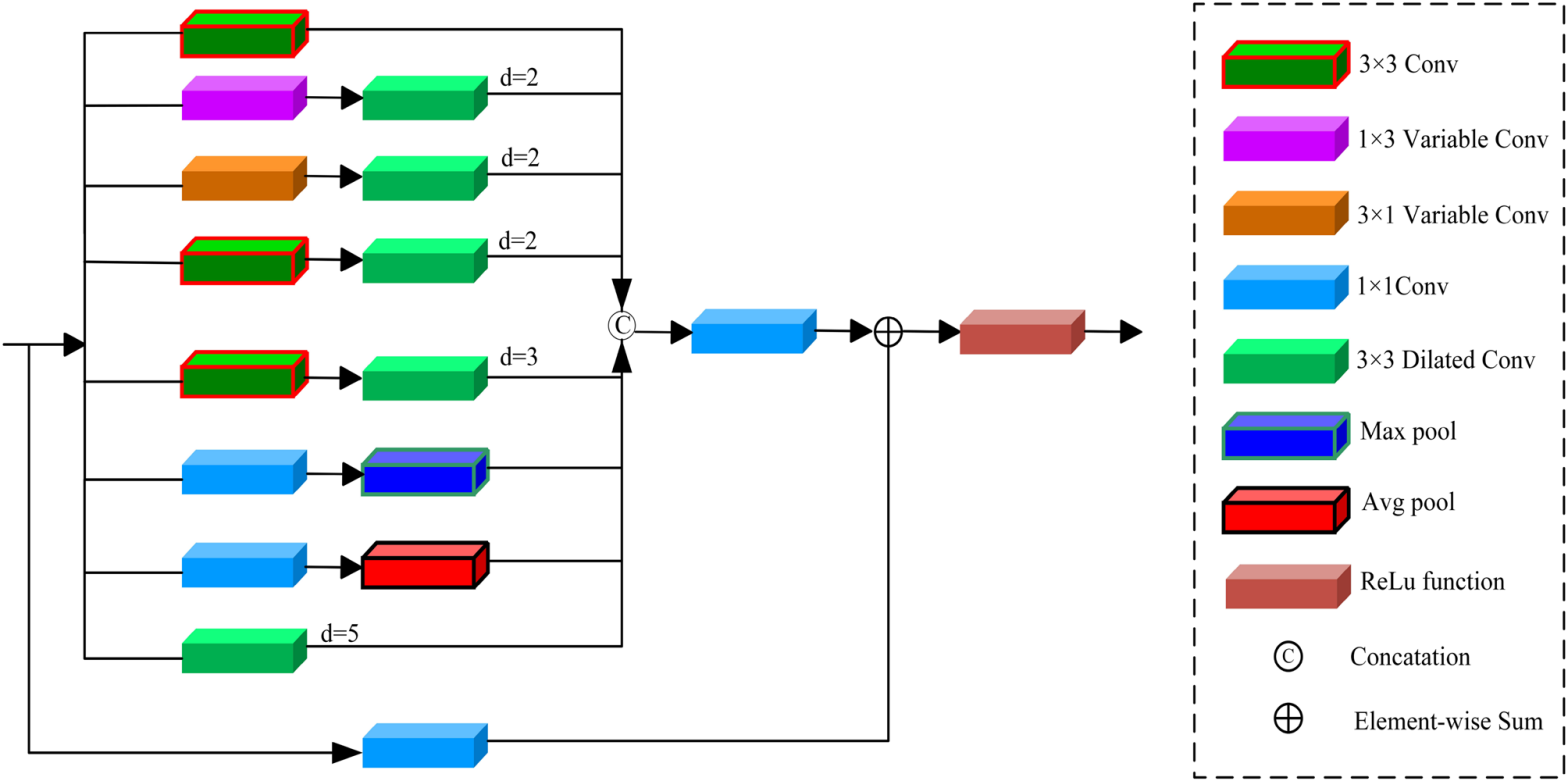
Proposed multi-scale feature extraction module.

In traditional standard convolutional feature extraction, limitations often arise due to the size of the receptive field, thereby overlooking various contextual information. To capture richer image details, we design a multi-scale feature extraction module and apply it to construct the basic feature extraction module in Fig. 1. Our designed multi-scale feature extraction module is depicted in Fig. 2. It comprises eight distinct branches. The first branch consists of a single 3$\times$3 convolutional layer. The second branch consists of a 1$\times$3 variable convolution followed by a dilated 3$\times$3 convolution with a dilation rate of 2. The third branch comprises a 3$\times$1 variable convolution followed by a dilated 3$\times$3 convolution with a dilation rate of 2. The fourth branch contains a 3$\times$3 convolutional layer followed by a dilated 3$\times$3 convolution with a dilation rate of 2. The fifth branch consists of a 3$\times$3 convolutional layer followed by a dilated 3$\times$3 convolution with a dilation rate of 3. The sixth branch comprises a 1$\times$1 convolutional layer followed by a max-pooling layer, significantly aggregating high-frequency information of low-light images. The seventh branch contains a 1$\times$1 convolutional layer followed by an average-pooling layer, smoothing overall features to highlight global feature information. The eighth branch consists of a dilated convolution with a dilation rate of 5. To reduce the parameters and computational load of the multi-branch model, the number of channels after the first convolution in each branch is reduced to 1/8 of the input channels. The outputs of these branches are concatenated, and the channel number of the feature map is restored to its original size through a 1$\times$1 convolutional layer. The receptive fields of these eight branches are 3$\times$3, 5$\times$7, 7$\times$5, 9$\times$9, 7$\times$7, 3$\times$3, 3$\times$3, and 11$\times$11, respectively. Additionally, we fuse the output features of the eight branches through concatenation and adjust the channel numbers with a 1$\times$1 convolution. Finally, we merge the fused feature map with the input feature map through element-wise addition to obtain the final output feature map.

**Figure 3:**
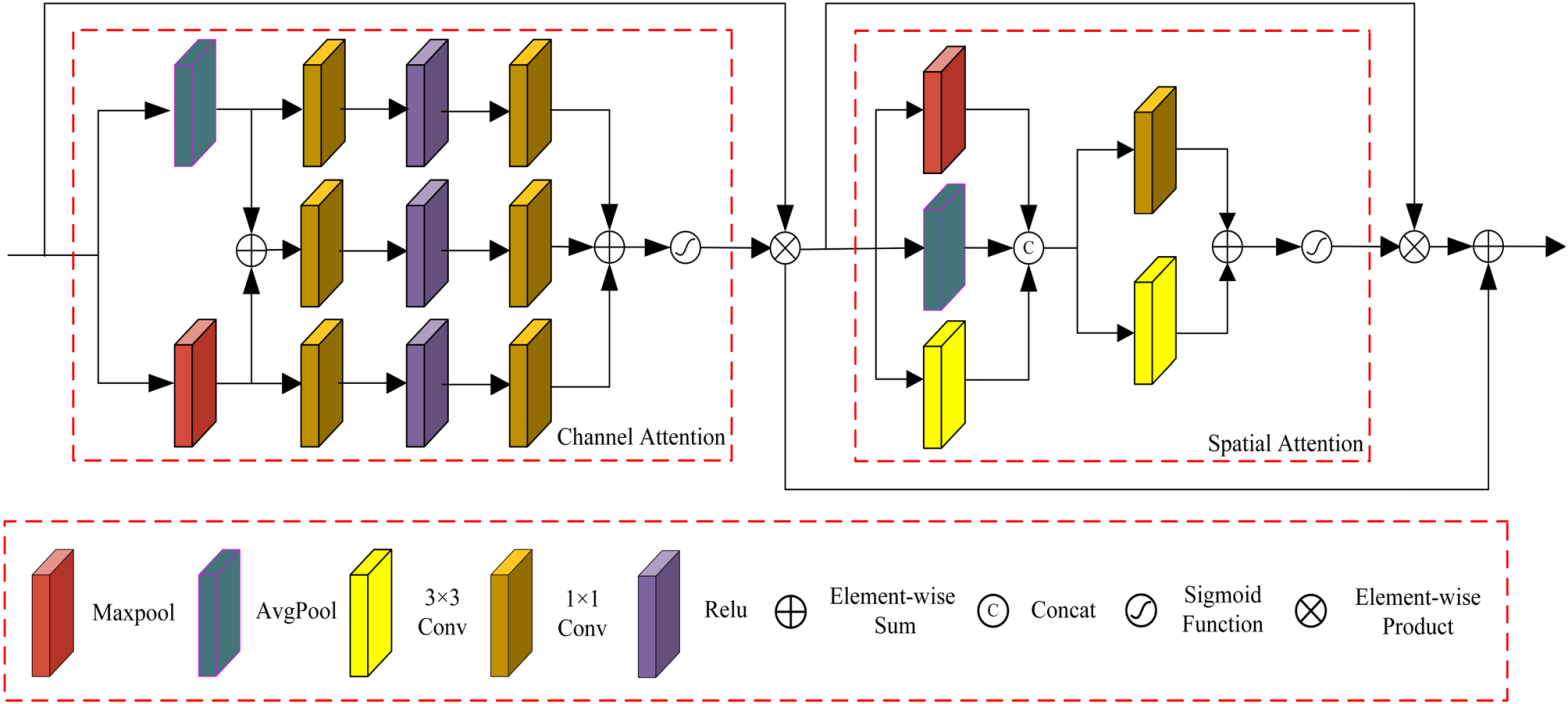
Proposed illumination attention module.

To reduce the impact of redundant feature information on enhancing low-light images, we design an illumination attention module as illustrated in Fig. 3. It is also part of the basic feature extraction module shown in Fig. 1. The illumination attention module comprises two parts: the channel attention module and the spatial attention module. The channel attention module consists of three branches. The first branch includes an average pooling followed by a fusion module. Average pooling captures background information of different channel feature maps across the entire image, such as overall brightness and color distribution. The fusion module consists of two 1$\times$1 convolutional layers with ReLU activation, which respectively reduce and increase the number of channels, preserving important information while reducing the parameters and complexity of the module. The second branch comprises a max pooling followed by a mixed module. Max pooling emphasizes the significant features in different channel feature maps. It is useful for identifying and extracting key information, such as object edges, textures, and image contrast. The third branch consists only of a fusion module. The structure of mixed modules in three branches is the same. The output feature map of the average pooling from the first branch and the output feature map of the max pooling from the second branch are element-wise added, yielding the input feature map for the third branch. After the element-wise addition of the output feature maps from the three branches, the feature map undergoes a Sigmoid function to obtain channel feature weights. Finally, the output feature map of the channel attention module is obtained by multiplying the channel feature weights with the input feature map of the channel attention module.

The spatial attention module consists of two concatenated parallel modules. The input feature map of the spatial attention module is the output feature map of the channel attention module. The first parallel module comprises max pooling, average pooling, and a 3$\times$3 convolution, serving to capture feature information between channels at different spatial positions. The 3$\times$3 convolution extracts conventional features. The number of channels for the output feature map in each parallel module is 1. After concatenating the output features from the three branches, the output feature map has three channels, which serve as the input feature map for the second parallel module in the spatial attention module. The second parallel module consists of parallel 1$\times$1 convolutions and a 3$\times$3 convolution. The two convolutions’ output feature maps are added element-wise, followed by a Sigmoid activation function, yielding spatial feature weights. Multiplying the spatial feature weights with the input feature map of the spatial attention module produces the output feature map of the spatial attention module. To allow the network to focus more on features such as illumination and texture details while reducing feature loss, we perform element-wise addition of the output feature map of the spatial attention module with the output feature map of the channel attention module to obtain the output feature map of the illumination attention module.

### Proposed discriminative network

**Figure 4:**
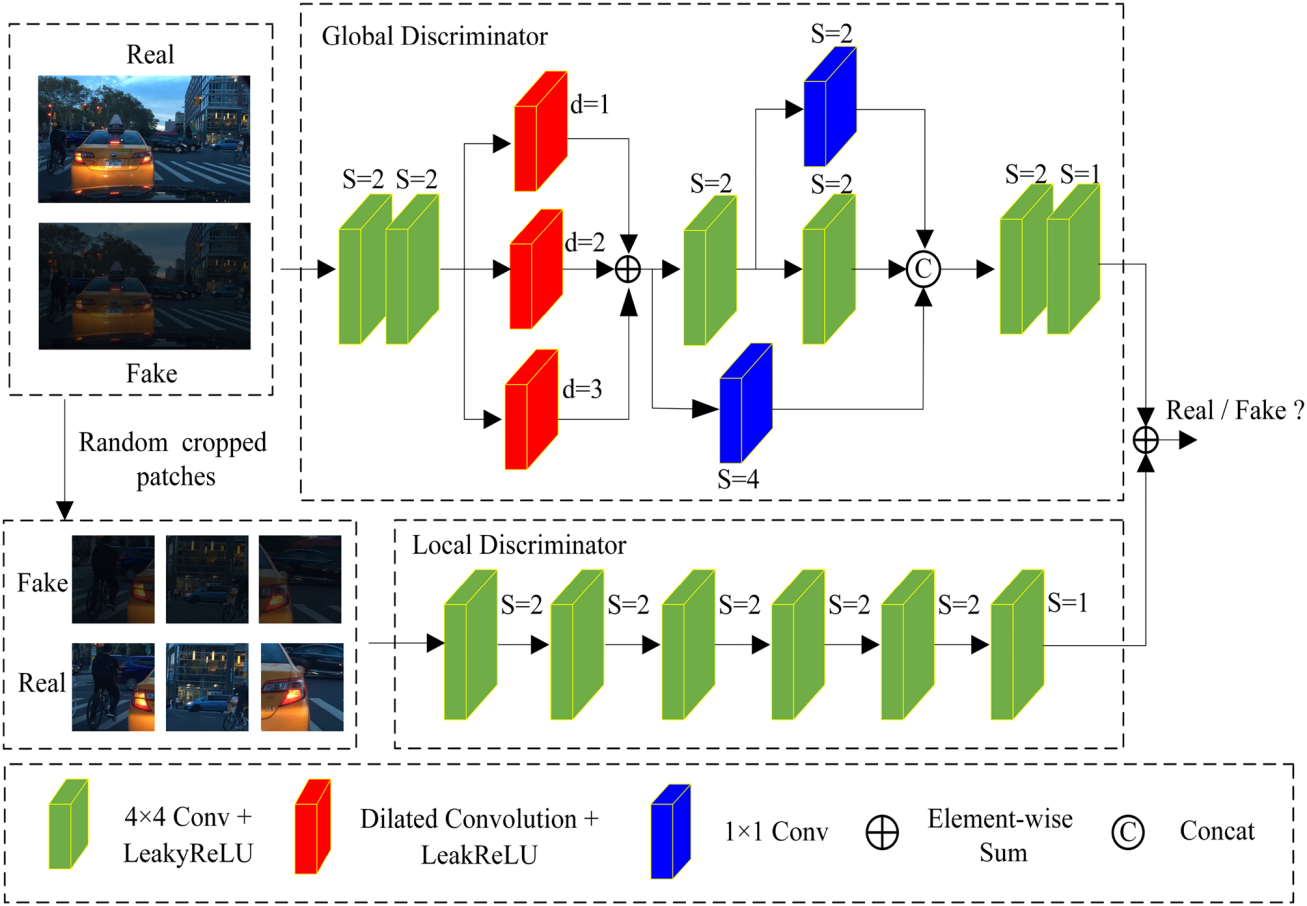
Proposed discriminative network.

In low-light images, bright and extremely dark areas differ significantly from the overall image features. Using a single global discriminative network alone makes it difficult to obtain accurate judgments. To enhance the performance of the discriminator, we designed a global-local discriminator based on the Markov discriminator, allowing the discriminator to better focus on both the background and local information of the image simultaneously. The designed global-local discriminator network is shown in Fig. 4. It consists of two parts: the global discriminator and the local discriminator. The input image for the global discriminator is the entire image, while the input image for the local discriminator is the image after random cropping of the entire image. Global discriminators primarily focus on the overall image, while local discriminators focus on the local areas of the image.

The global discriminator first utilizes two consecutive 4$\times$4 convolutions with LeakyReLU activation functions for initial feature extraction. Then, it employs a parallel convolution composed of three dilated convolutions with different dilation rates to extract features at different scales. Next, a residual network is used for further feature extraction. The main network of the residual network consists of two consecutive 4$\times$4 convolutions with LeakyReLU activation functions, and the branches of the residual network consist of two 1$\times$1 convolutions with strides of 2 and 4, respectively. A 4$\times$4 convolutional layer with a LeakyReLU activation function is utilized to extract features from the output feature map of the residual network. Finally, a 4$\times$4 convolution with the LeakyReLU activation function converts the extracted features into discrimination scores. The local discriminator consists of six consecutive 4$\times$4 convolutions with LeakyReLU activation functions. The first five 4$\times$4 convolutions are used to extract local feature information, and the last 4$\times$4 convolution is used to convert the extracted features into discrimination scores. The improved discriminator can better extract different contextual feature information while paying attention to more details, effectively improving its discrimination capability.

### Improved loss function

An improved loss function is proposed to better quantify the difference between the enhanced image and the normal illuminated image by introducing color loss and perceptual loss into the conventional loss function. The improved loss function can be represented as:

1 $$\begin{aligned} {L\mathrm{{oss = }}{L_{GAN}} + {L_{per}} + {L_{color}}} \end{aligned}$$

where $L_{GAN}$ represents the total loss of the generative adversarial network. It includes the global loss of the generative network, the local loss of the generative network, the global loss of the adversarial network, and the local loss of the adversarial network.$L_{per}$ denotes the perceptual loss, $L_{color}$ represents the color loss.

We use perceptual loss to measure the distance between the features of the generated images and those of the normal illuminated images, which is represented as follows:

2 $$\begin{aligned} {L_{per}}(x,z) = \frac{1}{{M \times W \times H}}\sum \limits _{s = 1}^M {\sum \limits _{i = 1}^W {\sum \limits _{j = 1}^H {\left\| {{\varphi _{s,i,j}}(G(z)) - {\varphi _{s,i,j}}(x)} \right\| _2^2} } } \end{aligned}$$

where $M$ is the number of images. $x$ represents the normal illuminated image. $G(z)$ represents the enhanced image. ${\varphi _{s,i,j}}$ denotes the value at position $(i,j)$ of the feature map extracted from the VGG16 network for the s-th image. $W$ and $H$ represent the width and height of the feature map, respectively.

The color loss is expressed as:

3 $$\begin{aligned} {L_{color}} = \frac{1}{{M \times N \times H \times W}}\sum \limits _{s = 1}^M {\sum \limits _{cth = 1}^N {\left| {\left( {\sum \limits _{i = 1}^H {\sum \limits _{j = 1}^W {{x_{s,i,j,cth}}} } - \sum \limits _{i = 1}^H {\sum \limits _{j = 1}^W {{z_{s,i,j,cth}}} } } \right) } \right| } } \end{aligned}$$

where $M$ is the number of images. $N$ is the number of channels for the input image ( $N=3$ ). $W$ and $H$ represent the width and height of the feature map, respectively. $cth$ represents the c-th channel of the sample. $x$ and $z$ represent the normal illuminated image and enhanced image, respectively. Color loss measures the difference between the enhanced and normal illumination images on each channel.

## Simulation and discussion

**Figure 5:**
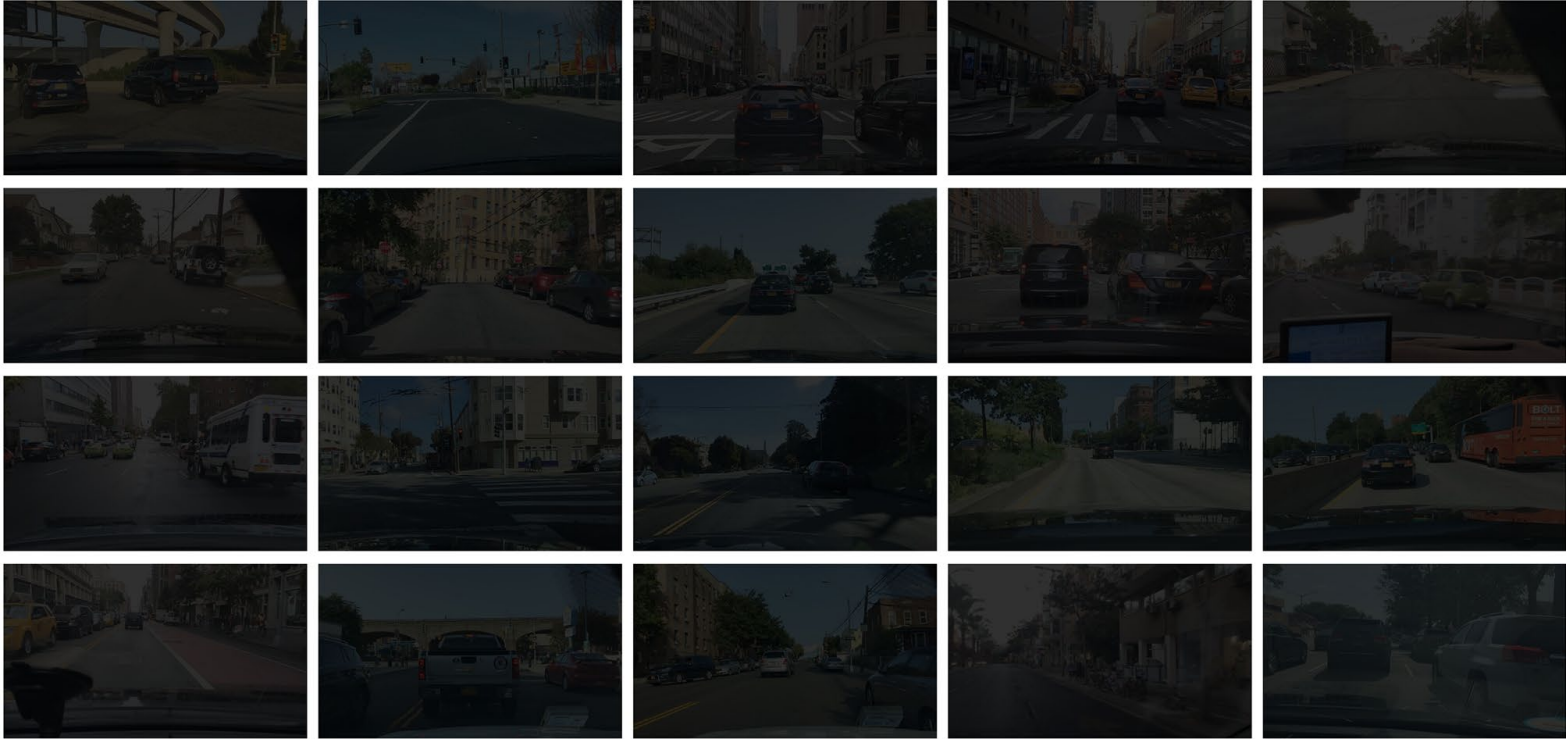
Partially synthesized low-light road images with a luminance adjustment index of 0.7.

To train the model, we randomly selected 10,000 normal illumination images from the BDD100K dataset and synthesized corresponding low-light images using an index transformation method. The formula for synthesizing low-light images is as follows:

4 $$\begin{aligned} {{I_{\mathrm{{out}}}}\mathrm{{ = k}} \times {{(}{I_{in}})^\alpha }} \end{aligned}$$

where $I_{in}$ represents the normal illumination image. $\textrm{k}$ represents the channel coefficient. $I_{\mathrm{{out}}}$ represents the generated low-light image. $\alpha$ represents the luminance adjustment index. This paper chooses the luminance adjustment index as a random number between 0.6 and 0.8 to simulate low-light images with varying luminance levels. To simulate the color distortion of low-light images, the channel coefficient $\textrm{k}$ is set as a random number between 0.95 and 1.05. Figure 5 shows synthesized low-light road images with a luminance adjustment index of 0.7. We paired the normal illumination images with their corresponding synthesized images, selecting 7000 image pairs for training and 3000 image pairs for testing. The proposed method, along with KinD, EnlightenGAN, Zero-DCE, Zero-DCE++, and SCI methods, are trained and tested on these image pairs. All models were trained using the same initial parameters, with the Adam optimizer, over 200 epochs, and with a learning rate of 0.001.

To quantitatively analyze the effectiveness of the algorithms, we used the following reference-based performance metrics: Peak Signal to Noise Ratio (PSNR)^38^, Structural Similarity (SSIM)^39^ and Learned Perceptual Image Patch Similarity (LPIPS)^40^ to evaluate the quality of the generated low-light images. For no-reference performance metrics, we used Natural Image Quality Evaluator (NIQE)^41^ and Meta Image Quality Assessment (MetaIQA)^42^ to assess the quality of enhanced images.

### Simulation on synthesized low-light images

**Figure 6:**
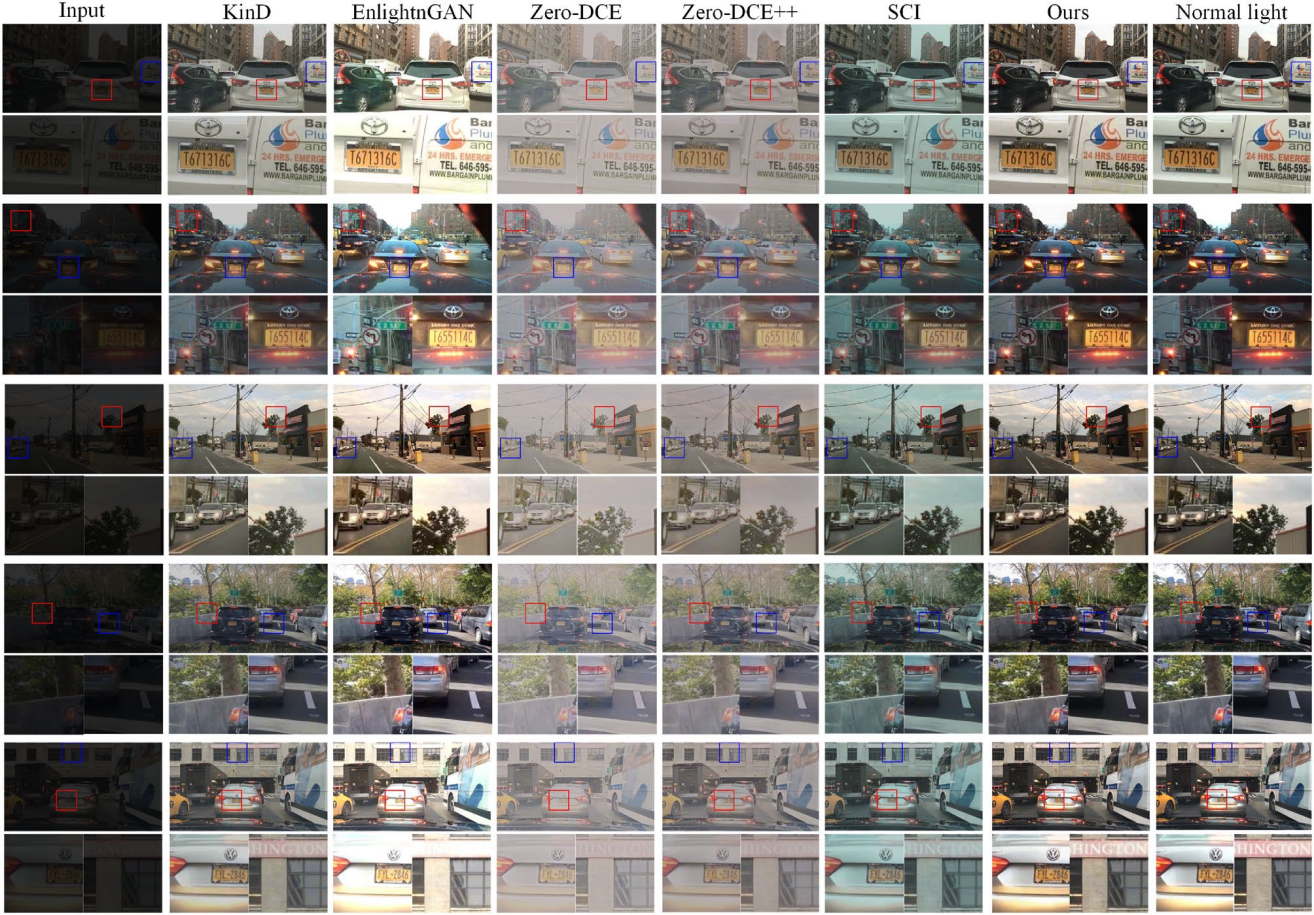
Low-light image, normal illumination image, enhanced images, and local magnified images.

We randomly selected five images from the test set as input images. The low-light, normal illumination, enhanced, and local magnified images are shown in Fig. 6. The first column represents synthesized low-light images. Columns two to seven show the enhanced images obtained by KinD, EnlightenGAN, Zero-DCE, Zero-DCE++, SCI, and our proposed method, respectively. The last column displays the original normal illumination images. From the images in columns four and five, it is evident that the images generated by Zero-DCE and Zero-DCE++ have lower clarity. Concomitantly, there is always a color deviation in the images generated by the SCI method. For the first and second images, the images enhanced by the EnlightenGAN method have slight overexposure in certain areas. The images enhanced by the KinD method and our proposed method are clearer and closer to normal illumination images. For the third image, the image enhanced by the KinD method retains the cloud colors well but lacks clarity. The image enhanced by the EnlightenGAN method preserves local details well but loses some cloud information due to higher brightness. The image enhanced by our algorithm retains detailed information better and is clearer. For the fourth image, the images generated by KinD, EnlightenGAN, and our method are similar in clarity, making it difficult to distinguish differences with the naked eye. For the fifth image, the image enhanced by the KinD method has lower saturation. The image enhanced by the EnlightenGAN method shows overexposure in the billboard, resulting in excessive brightness.

In summary, the images enhanced by the Zero-DCE and Zero-DCE++ methods have lower clarity, and the images enhanced by the SCI method consistently exhibit color deviation. Some images enhanced by EnlightenGAN show overexposure, and some enhanced by the KinD method have low saturation, resulting in less vibrant colors. Although the images enhanced by our method also exhibit some distortion, the distortion is minimal, and the images are clearer and closer to normal illumination images.

To more objectively evaluate the low-light image enhancement capabilities of various methods, we use all the low-light images from the test set as input images and PSNR, SSIM, and LPIPS to measure the quality of the enhanced images. The results are shown in Table 1. The average PSNR values for the images enhanced by the KinD method, EnlightenGAN method, Zero-DCE method, Zero-DCE++ method, SCI method, and our proposed method are 26.03, 27.31, 20.88, 21.84, 24.61, and 34.34, respectively. Our method has the highest PSNR value, followed by the EnlightenGAN and KinD methods. The average SSIM values for the images enhanced by the KinD method, EnlightenGAN method, Zero-DCE method, Zero-DCE++ method, SCI method, and our proposed method are 0.959, 0.965, 0.917, 0.922, 0.930, and 0.979, respectively. Our method has the highest SSIM value, followed by the EnlightenGAN and KinD methods. The average LPIPS values for the images enhanced by the KinD method, EnlightenGAN method, Zero-DCE method, Zero-DCE++ method, SCI method, and our proposed method are 0.084, 0.081, 0.104, 0.097, 0.090, and 0.061, respectively. Our method has the lowest LPIPS value, followed by the EnlightenGAN and KinD methods. In summary, our proposed method achieves the highest PSNR and SSIM values and the lowest LPIPS value, indicating that it has better low-light image enhancement capabilities than other methods.

**Table 1:** Performance comparison for synthesized low-light images .

	KinD	EnlightenGAN	Zero-DCE	Zero-DCE++	SCI	Ours
PSNR	26.03	27.31	20.88	21.84	24.61	34.34
SSIM	0.959	0.965	0.917	0.922	0.930	0.979
LPIPS	0.084	0.081	0.104	0.097	0.090	0.061

Table 2 presents the computational cost (FLOPs, floating point operations) and the number of parameters (Params) for each model, representing the time complexity and space complexity of the models, respectively. The FLOPs values for the KinD, EnlightenGAN, Zero-DCE, Zero-DCE++, SCI, and our method are 29.13G, 62.76G, 58.36G, 7.42G, 1.61G, and 51.43G, respectively. The Params values for the KinD, EnlightenGAN, Zero-DCE, Zero-DCE++, SCI, and our method are 8.54M, 8.63M, 0.10M, 0.02M, 0.23M, and 7.35M, respectively. The FLOPs value of our method is higher than that of the KinD, Zero-DCE++, and SCI methods and lower than that of the EnlightenGAN and Zero-DCE methods. For real-time applications of our proposed method, the computing device needs to have strong computational capabilities.

**Table 2:** The FLOPs and Params values of different methods.

	KinD	EnlightenGAN	Zero-DCE	Zero-DCE++	SCI	Ours
FLOPs	29.13	62.76	58.36	7.42	1.61	51.43
Params	8.54	8.63	0.10	0.02	0.23	7.35

### Simulation on the low-light images with different brightness levels

**Figure 7:**
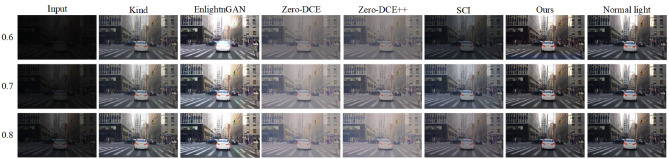
Low-light images with different brightness levels and enhanced images.

We set different brightness adjustment exponents for images to test the enhancement capabilities of various methods on low-light images with different brightness levels. The brightness adjustment exponents are set to 0.6, 0.7, and 0.8, representing extremely dark, dim, and faint light conditions, respectively. The low-light images with different brightness levels and the enhanced images are shown in Fig. 7. The first column represents synthesized low-light images with different brightness levels. Columns two to seven show the enhanced images obtained by KinD, EnlightenGAN, Zero-DCE, Zero-DCE++, SCI, and our proposed method, respectively. The last column displays the original normal illumination images. Figure 7 shows that as the adjustment exponent decreases, the quality of the images enhanced by all methods declines. When the brightness adjustment exponent is 0.6, the images enhanced by Zero-DCE and Zero-DCE++ exhibit blurriness, the images enhanced by SCI and KinD suffer from underexposure, and the images enhanced by EnlightenGAN have noticeable local overexposure issues. The images enhanced by our method maintain higher clarity and are closer to real images.

The performance metrics of different methods for images with different brightness levels are shown in Table 3. As seen in Table 3, our method consistently achieves higher PSNR and SSIM values and lower LPIPS values across different brightness adjustment exponents compared to other methods. This indicates that our method has better image enhancement capabilities for low-light images with varying brightness levels.

**Table 3:** Performance comparison for low-light images with different brightness levels.

	Brightness adjustment exponent	KinD	EnlightenGAN	Zero-DCE	Zero-DCE++	SCI	Ours
PSNR	0.6	23.97	26.23	18.55	19.13	23.87	33.38
	0.7	25.42	28.45	20.49	22.38	25.46	34.15
	0.8	26.10	29.69	21.64	22.45	26.10	34.52
	Average	25.16	28.12	20.23	21.32	25.14	**34.02**
SSIM	0.6	0.911	0.887	0.876	0.896	0.922	0.949
	0.7	0.943	0.972	0.910	0.934	0.918	0.963
	0.8	0.952	0.966	0.925	0.927	0.945	0.978
	Average	0.935	0.941	0.903	0.919	0.928	**0.963**
LPIPS	0.6	0.088	0.081	0.106	0.098	0.084	0.064
	0.7	0.083	0.075	0.101	0.096	0.082	0.062
	0.8	0.082	0.072	0.098	0.092	0.083	0.058
	Average	0.084	0.076	0.101	0.095	0.083	**0.061**

Significant values are in bold.

### Simulation on the real low-light image

**Figure 8:**
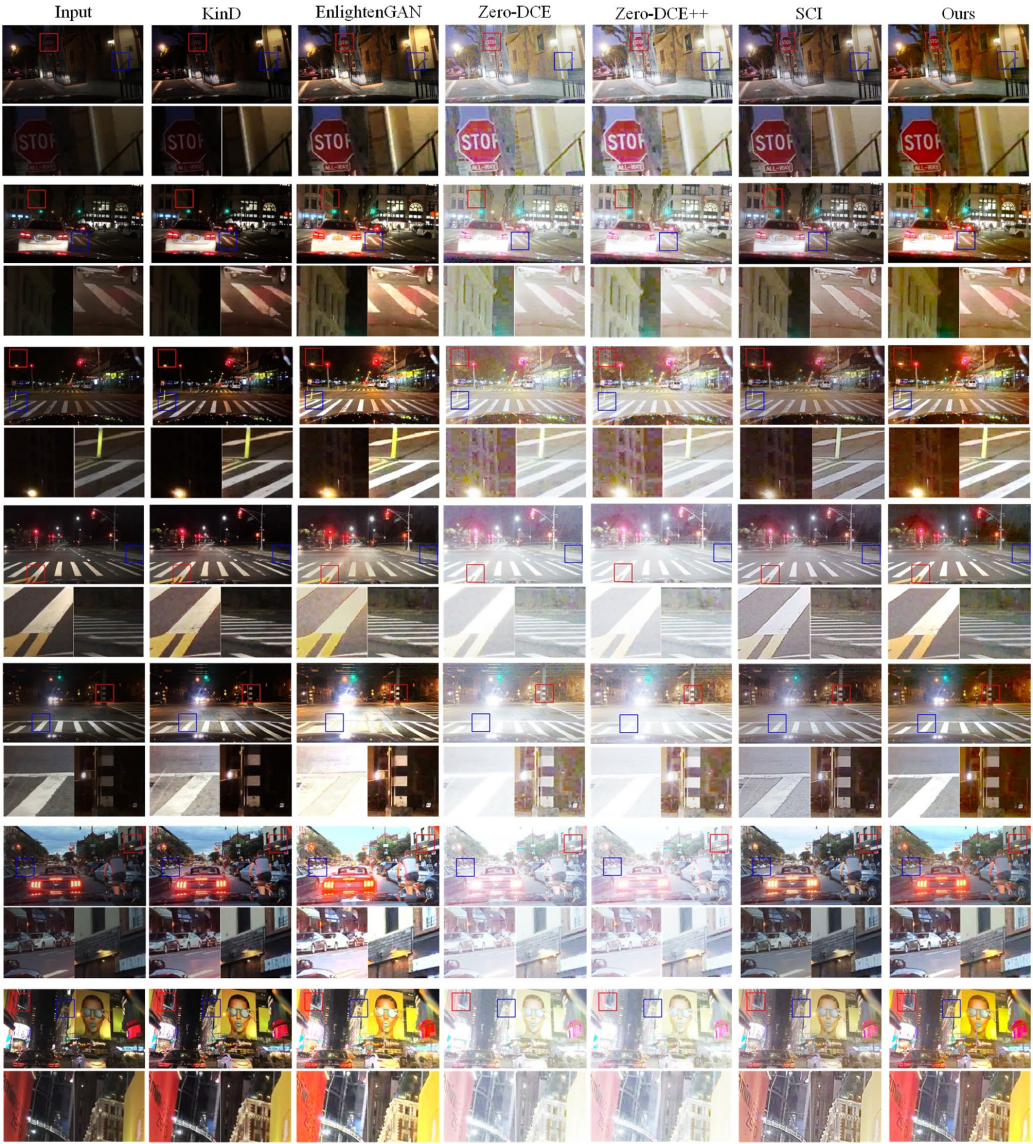
Real Low-light images, enhanced images, and local magnified images.

To test the enhancement capabilities of various methods on real low-light images, we selected seven real low-light images from the internet and enhanced them using different methods. Figure 8 shows the original and enhanced images. In Fig. 8, the first column shows the real low-light images and their corresponding magnified local images. Columns two to seven display the enhanced images generated by the KinD method, EnlightenGAN method, Zero-DCE method, Zero-DCE++ method, SCI method, and our proposed method, respectively. For the real images in the first column, the first and second images are extremely dark night images, the third to fifth images are darker night images, the sixth image is an afternoon scene, and the seventh image represents a mildly non-uniform night image. All images enhanced by Zero-DCE and Zero-DCE++ methods exhibit overexposure issues. The first, second, and third images enhanced by the KinD algorithm lack sufficient brightness, with underexposure issues noticeable in the magnified local images on the left. The third image enhanced by the EnlightenGAN algorithm also lacks sufficient brightness, with underexposure issues in the left-magnified local image. In addition, the fifth image is locally too bright and has an overexposure problem in the local zoomed-in image on the left side and a color distortion problem in the local zoomed-in image on the right side. The fourth image enhanced by the SCI algorithm exhibits some local color distortion, especially with yellow color distortion on the zebra crossing in the left-magnified local image. The afternoon image in the sixth row has both daytime and dark scenes. Our method enhances the backlit dark areas better compared to other methods and restores the original colors the best. For the mildly non-uniform night image in the seventh row, the KinD method is inadequate in enhancing the dark areas of the seventh image. The EnlightenGAN method causes distortion in the seventh image, losing significant detail. The SCI method still shows color deviation in the seventh image. Our method significantly enhances the image details of the tree trunk in the first image and restores the true color of the dark wall in the second image. In the fourth and fifth images, our enhancement method does not cause overexposure to the streetlights. For the mildly non-uniform night image in the sixth image, our method enhances the details in the dark areas without causing overexposure in the bright areas. Therefore, our method can effectively enhance night scene images with varying degrees of darkness. However, for highly non-uniform brightness images, our method results in localized overexposure and distortion in the enhanced images.

To more objectively evaluate the performance of the low-light image enhancement methods, we used NIQE and MetaIQA to measure the quality of the enhanced images. The results are shown in Table 4. From Table 4, it is evident that our method achieves the lowest NIQE and the highest MetaIQA, indicating that the enhanced low-light images generated by our method have better quality and visual appeal. This also demonstrates that our method performs well in enhancing real low-light images.

**Table 4:** Performance comparison for real low-light images.

	KinD	EnlightenGAN	Zero-DCE	Zero-DCE++	SCI	Ours
NIQE	4.942	4.814	5.569	5.452	5.181	4.368
MetaIQA	0.273	0.248	0.216	0.227	0.313	0.341

### Ablation study

To verify the effectiveness of the different modules proposed in this paper, we test the performance of the algorithm after individually removing the Illumination Attention Module (No-LAM), the Multi-Scale Feature Extraction Module (No-MFEM), using a conventional Markov Discriminator Network (No-GFRM), and using a conventional loss function (No-CLoss). The test results are shown in Table 5. The PSNR values for No-LAM, No-MFEM, No-GFRM, No-CLoss, and the complete algorithm are 28.60, 30.28, 32.47, 33.55, and 34.34, respectively. The SSIM values are 0.949, 0.953, 0.968, 0.975, and 0.979, respectively. The LPIPS values are 0.083, 0.076, 0.072, 0.066, and 0.061, respectively. The performance metrics vary when different modules are removed, validating the effectiveness of the proposed modules. Additionally, removing the Illumination Attention Module resulted in the highest PSNR and SSIM values and the lowest LPIPS value, indicating that the Illumination Attention Module contributes most significantly to the overall performance improvement of the algorithm.

**Table 5:** Performance comparison for removing proposed modules.

	No-LAM	No-MFEM	No-GFRM	No-CLoss	Ours
PSNR	28.60	30.28	32.47	33.55	34.34
SSIM	0.949	0.953	0.968	0.975	0.979
LPIPS	0.083	0.076	0.072	0.066	0.061

### User study

**Figure 9:**
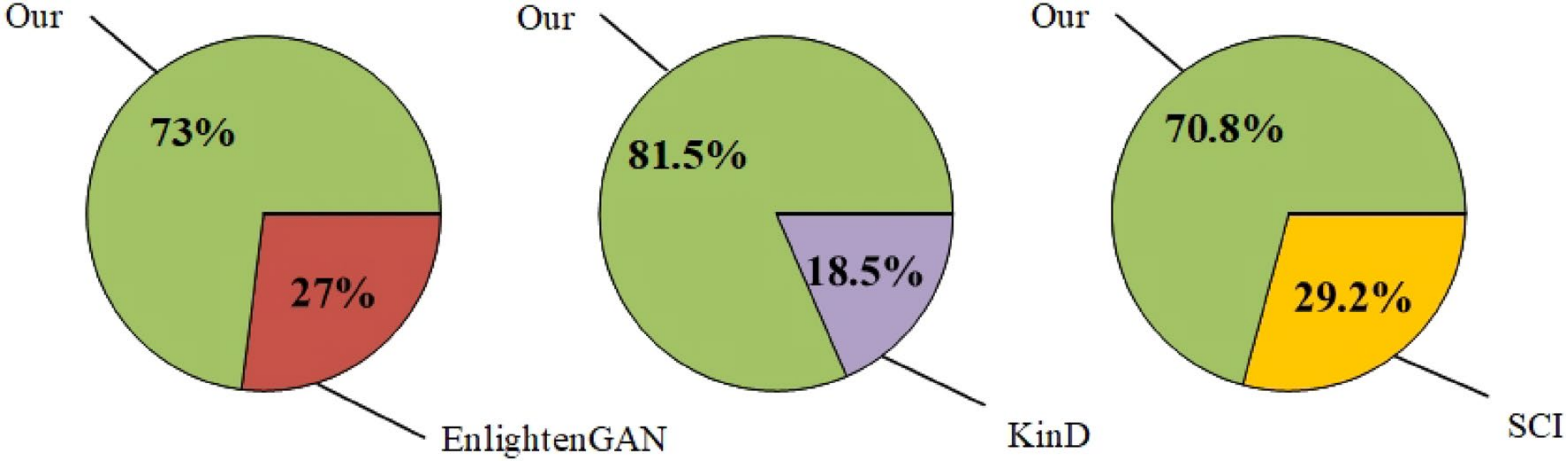
User study results on real night-time images.

To further verify the effectiveness of our proposed method, we conducted a user study. Due to the significantly poorer performance of the Zero-DCE and Zero-DCE++ methods compared to ours, we only compared KinD, EnlightenGAN, SCI, and our method. A total of 30 volunteers participated in this user study. We randomly selected 100 real low-light images from the internet and obtained the enhanced images using KinD, EnlightenGAN, SCI, and our method. We created three tests by pairing our method with each of the other three methods. Each test contained 100 sets of images. Each set displayed two images side by side: one enhanced by the comparative method and the other by our method. Volunteers randomly selected 40 sets from each test and chose the visually more satisfactory (more natural) image from each set. We collected the results of each volunteer’s choices and calculated the satisfaction for each of the three tests. The average satisfaction across all volunteers was then computed to obtain the final satisfaction rate. The final results are shown in Fig. 9. As can be seen, our method demonstrates better visual performance compared to KinD, EnlightenGAN, and SCI.

## Conclusion

This paper proposes a generative adversarial network for enhancing low-light images. The network consists of a generator and a discriminator. In the generator network, we design a multi-scale feature extraction module and an illumination attention module, which are used to construct the generator. In the discriminator network, we propose a dual-discriminator structure composed of a global discriminator and a local discriminator to enhance the discrimination capability of the discriminator network. Additionally, we introduce an improved loss function by incorporating color loss and perceptual loss into the conventional loss function, thereby improving the measurement of image distortion. We use the synthesized low-light images and real low-light images to test the methods, respectively. For the synthesized low-light images, the images enhanced by our method are closer to normal illumination images compared to other methods and also have the highest PSNR and SSIM values and the lowest LPIPS values. For the real low-light images, the images enhanced by our method have the lowest NIQE and the highest MetaIQA and exhibit better clarity and visual effects than other methods. Simulation results show that our method performs better in enhancing the synthesized low-light images and real low-light images than other methods.

However, this paper’s proposed network only applies to low-light image enhancement in road scenes. Additionally, using only a generative adversarial network model for image enhancement may have certain limitations, and the problem of image distortion cannot be completely resolved. In the future, we plan to integrate the Retinex theory or other illumination models, incorporating physical knowledge into the deep learning framework, and focusing on making the model more lightweight. This will enhance the model’s understanding of illumination and reflection characteristics, making it faster and improving network performance and operational efficiency. Alternatively, we may consider using edge filtering techniques and incorporating information from other modalities to reduce detail loss during the enhancement process, thereby further addressing the distortion issues post-enhancement.

## Data Availability

The dataset used in this article is publicly available and can be accessed at http://bdd-data.berkeley.edu/
